# Reason and Instinct

**Published:** 1859-02

**Authors:** W. W. Hall


EXTRACTS FROM HEALTH AND DISEASE, BY DR. W. W. HALL.

### REASON AND INSTINCT.

THE power which sets all stars and suns in motion, ordained that it should be kept in continuance by inherent properties; we call it gravitation. That same power started the complex machinery of corporeal man, and endowed it with regulations for continuance to the full term of animal life, and we call it instinct.

The irresponsible brute has no other guide to health, than that of instinct—it is in a measure absolutely despotic, and can not be readily contravened.

By blindly and implicitly following this instinct, the birds of the air, the fish in the sea, and four-footed beasts and creeping things live in health, propagate their kind, and die in old age, unless they perish by accident or by the warfares which they wage against one another, living, too, from age to age without any deterioration of condition or constitution; for the whale of the sea, the lion of the desert, the fawn of the prairie, are what they were a thousand years ago; and that they have not populated the globe is because they prey on one another, and man in every age has lifted against them an exterminating arm. Man has instinct in common with the lower races of animal existence, to enable him to live in health, to resist disease; but he has in addition a higher and a nobler guide—it is Reason. Why he should have been endowed with this additional safeguard, is found in the fact, that the brute creation are to be used for temporary purposes, and at death their light goes out forever, but man is designed for an immortal existence, of which the present life is the mere threshhold. He is destined to occupy a higher sphere, and a higher still, until in the progress of ages, he passes by angelic nature; rising yet, archangels fall before him, and leaving these beneath, and behind him, the regenerated soul stands in the presence of the Deity, and basks forever in the sunshine of his glory.

Considering then, that such is his ultimate destination, it is no wonder that in his wise benevolence, the great Maker of us all should have vouchsafed to the creature man, the double safe-guard of instinct, and of a diviner reason; that by the aid and application of both, his life might be protected, and protracted too, under circumstances of the highest advantage and most extended continuance, in order to afford him the fullest opportunity of preparing himself for a destiny so exalted, and for a duration of ceaseless ages.

